# DNA Nanotechnology-Enabled Precise Regulation of Nanozymes and Their Applications

**DOI:** 10.34133/research.1293

**Published:** 2026-06-11

**Authors:** Zishuo Liu, Yuanbo Zhao, Haoxiang Chen, Xin Fu, Jinyue Fan, Lingyan Lu, Zhiwen Lyu, Honghui Cai, Ziming Wang, Chao Zhang, Fan Xiao, Yao Xiao

**Affiliations:** ^1^ Department of Neurosurgery, Zhujiang Hospital, Southern Medical University, Guangzhou 510282, P.R. China.; ^2^ Department of Oncology, Zhujiang Hospital, Southern Medical University, Guangzhou 510282, P.R. China.; ^3^ Department of Respiratory and Critical Care Medicine, Sir Run Run Shaw Hospital, Zhejiang University School of Medicine, Hangzhou, Zhejiang 310016, P.R. China.; ^4^ Clinical Skills Training Center, Zhujiang Hospital, Southern Medical University, Guangzhou 510282, P.R. China.

## Abstract

Nanozymes overcome key limitations of natural enzymes, including poor environmental tolerance, high production costs, and batch-to-batch variability, with their enhanced physicochemical stability and controllable catalytic performance, thus enabling applications in biocatalysis and disease theranostics. However, their practical applications remain limited by insufficient catalytic efficiency, inadequate target specificity, and restricted programmable dynamic adaptability. DNA nanotechnology offers a versatile and biocompatible framework for the precise regulation of nanozymes due to its sequence-defined programmability and precise molecular recognition capability. This approach improves catalytic performance while maintaining excellent biocompatibility and low immunogenicity. This review systematically summarizes the latest advances in DNA nanotechnology-mediated precise regulation of nanozymes, with particular emphasis on the mechanistic basis of 6 regulatory modes. We further examine the representative applications of these DNA–nanozyme hybrid systems in disease therapy, biomedical sensing, and environmental monitoring, and outline the key challenges and future directions toward clinical and industrial translation.

## Introduction

Natural enzymes serve as highly specific biocatalysts central to metabolic regulation, which accelerate metabolic processes by lowering activation energies and act as critical regulators in biological systems [1]. Nevertheless, their practical applications are often limited by intrinsic instability [2], high production costs, and sensitivity to environmental fluctuations such as pH and temperature, which collectively result in poor long-term stability [1,2]. Since the seminal discovery of the intrinsic peroxidase-like activity of magnetic nanoparticles by Gao et al. [3], nanozymes have emerged as robust alternatives to natural enzymes. By overturning the traditional view that inorganic nanomaterials are “biologically inert” [4], nanozymes combine the stability of inorganic cores with enzyme-mimetic catalysis and scalable production [5], enabling broad applications in biosensing, disease theranostics, and environmental monitoring [6,7].

Despite these advantages, the clinical and industrial translation of nanozymes faces long-standing challenges regarding catalytic efficiency and specificity. Unlike the sophisticated active pockets of natural enzymes [6], nanozymes typically rely on surface unsaturated coordination bonds or specific crystal planes for catalysis [5], resulting in catalytic activity that is often far lower than their natural counterparts [6]. This structural rigidity limits the dynamic conformational changes required for substrate recognition. As a result, substrate affinity is often compromised, and programmable regulatory flexibility remains limited. Furthermore, nonspecific surface adsorption in complex biological matrices often covers active sites and leads to weak substrate specificity [6,8], alongside potential issues with biocompatibility [4].

To address these limitations, DNA nanotechnology provides a versatile and robust engineering platform by using DNA molecules as programmable nanoscale building blocks [9]. Using the strict Watson–Crick base-pairing mechanism, DNA nanotechnology enables the construction of functional nanostructures with precise geometries [10,11]. Over the past decades, this field has evolved from early tile-based assemblies to complex DNA origami structures [9,12], offering distinct advantages including high programmability, precise molecular targeting [13], excellent biocompatibility [14], and stimulus-responsive dynamic behavior [15]. Compared with conventional nanomaterial scaffolds such as polymers, metal–organic frameworks, and inorganic nanocarriers, DNA nanotechnology offers unique advantages in nanozyme regulation. Its sequence-defined programmability enables precise spatial positioning and stoichiometric control of catalytic units at the nanoscale, while its inherent molecular recognition capability allows highly specific substrate targeting and responsive activation [16,17]. In addition, the dynamic and reversible structural transitions of DNA provide opportunities for constructing stimulus-responsive nanozyme systems, which are difficult to achieve using traditional materials [18]. These features collectively establish DNA nanotechnology as a superior and versatile scaffold for the precise regulation of nanozyme activity. The integration of DNA nanostructures with nanozymes acts as a rigid-flexible coupling strategy, which not only improves the accessibility of active sites through targeted substrate recognition [19] but also provides intelligent regulatory capabilities upon the inorganic cores. Consequently, these DNA-regulated nanozymes achieve switch-like control over catalytic activity and facilitate cascade reactions [20], markedly broadening their applications in biomedical sensing and precision medicine.

In this review, we discuss recent advances in DNA nanotechnology-mediated nanozyme regulation and systematically analyze the molecular mechanisms underlying 6 major regulatory strategies. We specifically focus on how DNA nanostructures modulate the catalytic microenvironment and substrate interaction of nanozymes. Finally, we highlight key challenges and outline future research directions for the clinical and industrial translation of DNA-regulated nanozymes.

## Precise Regulation of Nanozymes via DNA Nanotechnology

**Fig. 1.**
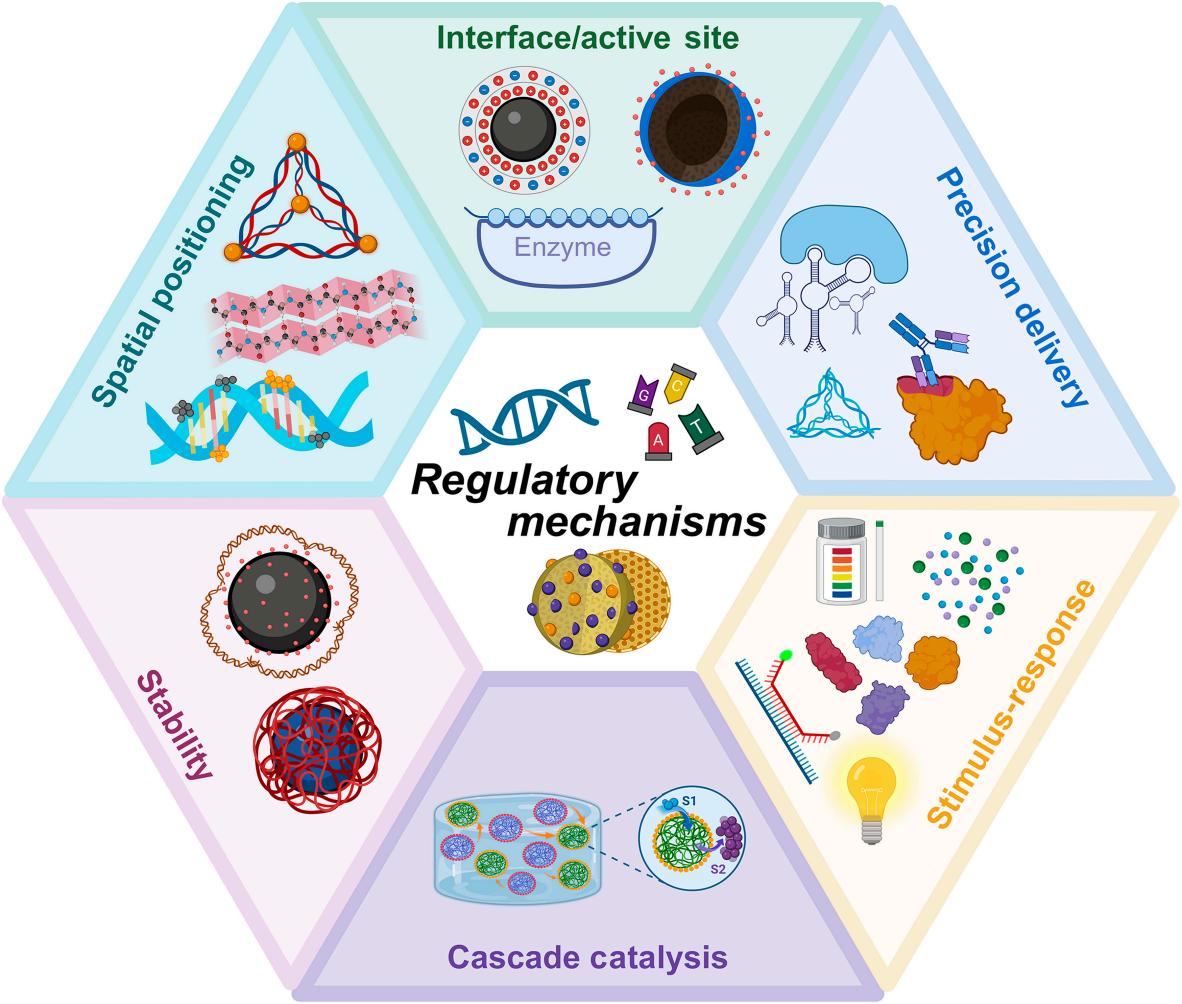
Overview of regulatory mechanisms of nanozymes enabled by DNA nanotechnology. Created with BioRender.com.

The catalytic performance of nanozymes is fundamentally determined by their spatial organization, interfacial properties, in vivo delivery efficiency, dynamic controllability, cascade reaction efficiency, and structural stability, as summarized in Fig. 1. Using its base-pairing programmability and nanoscale precision, DNA nanotechnology provides a molecularly tunable toolbox to optimize the above 6 key dimensions of nanozymes, breaking through the inherent bottlenecks of conventional nanozymes. In this section, we systematically categorize DNA-mediated nanozyme regulation into 6 key modes and describe their molecular mechanisms, representative strategies, and latest research advances. To improve readability, representative studies are discussed below using their principal reported performance indicators, while direct cross-system comparison should be interpreted with caution. Additionally, a comparative summary of the 6 regulatory modes is provided in Table 1 to highlight their distinct mechanistic principles, key features, and representative strategies.

**Table 1.** Comparation of 6 DNA nanotechnology-enabled regulatory modes for nanozyme regulation

Regulatory mode	Core mechanism	Key features	Representative strategies	Reference
Spatial positioning regulation	Programmable DNA scaffolds organize nanozymes with defined spacing and geometry	Ordered arrangement; optimized microenvironment; improved electron transfer	DNA nanoribbons; tetrahedral/FNA scaffolds; ssDNA-templated growth	[25,26,28,33]
Interface and active site regulation	DNA modulates substrate access and catalytic centers through interfacial engineering and coordination	Tunable binding; enhanced activity/selectivity; increased active site exposure	DNA coating; DNA-directed PtNP growth; base–metal coordination; DNA nanocages	[38,42,46,49]
Precision delivery regulation	DNA enables targeted delivery through molecular recognition and structural control	Improved biodistribution; enhanced cellular uptake and targeting specificity	Aptamer targeting; DNA tetrahedra; RCA-derived nanostructures; antibody conjugation	[30,32,41,53,59]
Stimulus-responsive regulation	Stimuli-triggered DNA conformational switching for reversible catalytic activation	Dynamic ON/OFF control; spatiotemporal regulation; multi-stimuli responsiveness	pH-responsive i-motif; metal-ion coordination; strand displacement; light-responsive systems	[49,59,66,70,73,81]
Cascade catalytic regulation	Spatially confined sequential catalysis enabling substrate channeling	Multistep catalysis; reduced diffusion loss; signal amplification	DNA-scaffolded cascades; nanozyme–DNAzyme coupling; HCR/RCA amplification	[31,39,59,81]
Stability regulation	DNA forms protective shells to preserve dispersion and catalytic function under stress	Anti-aggregation; resistance to biofouling and nucleases; improved durability	DNA corona; nanoflower encapsulation; Au–S anchoring; steric/electrostatic stabilization	[25,53,59,93,95]

### Spatial positioning regulation

DNA nanotechnology provides a structurally addressable framework for organizing nanozymes with defined distances, orientations, and valencies, thereby enabling spatially controlled regulation of catalytic behavior. Spatial positioning regulation uses the programmable self-assembly of DNA to engineer nanostructures with precisely defined geometries and binding sites [21]. Spatial positioning regulation refers to the rational design of static or predefined spatial architectures to control nanozyme distribution, thereby optimizing catalytic microenvironments and improving reaction efficiency. These strategies can be implemented across multiple structural dimensions, ranging from 1-dimensional (1D) DNA nanoribbons (DNRs) to 3-dimensional framework nucleic acids (FNAs) and sequence-defined single-stranded DNA (ssDNA) templates. Acting as structural rigid scaffolds, these structures control the distribution and interparticle spacing of nanozymes, thereby preventing the aggregation-induced burial of active sites [22,23]. The ordered spatial arrangement of nanozymes regulates local catalytic microenvironments by controlling interparticle distance, substrate accessibility, and electron transfer pathways [24,25]. This organization improves catalytic efficiency and reaction kinetics compared with randomly distributed nanozyme systems [26].

#### Spatial positioning regulation mediated by DNRs

**Fig. 2.**
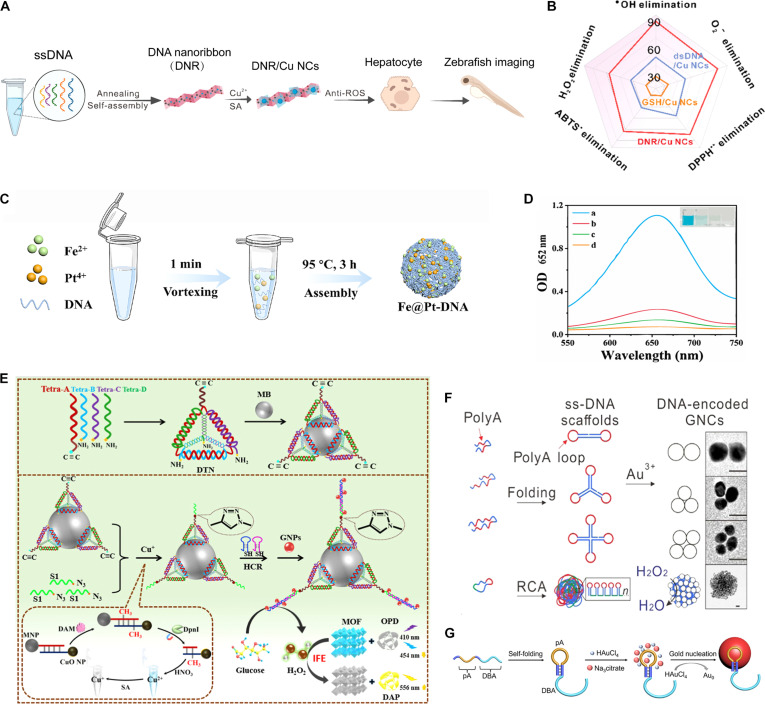
DNA nanotechnology-mediated spatial localization for regulating nanozyme activity. (A) DNA nanoribbon (DNR)-templated synthesis of Cu nanoclusters for anti-ROS therapy and zebrafish imaging. (B) Comparison of ROS scavenging activities of DNR/Cu NCs, GSH/Cu NCs, and free Cu NCs. (A and B) Reproduced from Ref. [28]. Copyright 2025 Elsevier Inc. All rights reserved. (C) DNA-mediated co-assembly of Fe@Pt-DNA bimetallic nanozymes with spatially separated active sites. (D) Peroxidase-like activity of Fe@Pt-DNA nanozymes compared with controls. (C and D) Reproduced from Ref. [49]. Copyright 2024 Elsevier B.V. All rights reserved. (E) DNA tetrahedron (DTN)-based spatial organization of multi-enzyme cascade nanozymes. Reproduced from Ref. [31]. Copyright 2024 The Royal Society of Chemistry. (F) PolyA DNA scaffold-directed synthesis of uniform gold nanoclusters. (G) ssDNA-directed nucleation and growth of Au nanoclusters. (F and G) Reproduced from Ref. [125]. Copyright 2024 Elsevier B.V. All rights reserved.

DNRs provide programmable, 1D templates for the anisotropic growth and organization of metal nanoclusters [27]. Li et al. [28] used pre-engineered DNRs to recruit copper ions along the phosphate backbone via electrostatic interactions, enabling the in situ formation of periodically arranged Cu nanoclusters (Fig. 2A and B). In contrast to randomly mixed systems, this scaffold-guided assembly enables a more uniform distribution of catalytic sites along the nanoribbon framework. Functional assays showed that the resulting architecture shows exceptional antioxidant activity mimicking superoxide dismutase (SOD) and glutathione peroxidase, significantly outperforming free Cu nanoclusters synthesized without templates. The enhanced catalytic performance is attributed to several factors, primarily the prevention of nanocluster agglomeration to maximize active site exposure, the optimization of electron transfer pathways along the 1D ordered structure, and the biological synergy activated by the DNA backbone.

In addition to serving as spatial templates, DNA strands can also participate directly in metal coordination and nucleation processes. Through specific coordination between nucleobases and metal ions (e.g., iron and platinum), ssDNA facilitates the spatially confined co-reduction and nucleation of bimetallic nanozymes, resulting in superior catalytic efficiency compared to monometallic systems (Fig. 2C and D) [29].

#### Spatial positioning regulation mediated by FNAs

FNAs, characterized by their distinct 3D geometries and spatial addressability [10], enable the precise stoichiometry and orientation of nanozymes [30]. For instance, tetrahedral DNA nanostructures serve as rigid scaffolds with defined vertices and excellent cellular permeability, making them robust platforms for ordered assembly (Fig. 2E). Cao et al. [31] used these pre-engineered frameworks (Alk-DTN) to assemble gold nanoparticles (AuNPs) in an ordered manner, effectively transitioning nanozymes from a stochastic distribution to ordered clusters. Such spatial reconfiguration significantly enhances catalytic turnover and signal amplification capabilities [6,32]. Notably, the integration of FNAs provides additional functions beyond catalysis, including targeted delivery and logic-gated activation. For example, fusing DNA logic gates with tetrahedral structures enables the conditional release of nanozymes upon recognizing specific cancer cell membrane markers [33]. These systems have thus become a cornerstone in the development of ratiometric fluorescence sensors [31], electrochemical devices [34], and multicolor colorimetric assays [35].

#### Spatial positioning regulation mediated by ssDNA sequences

At the molecular level, ssDNA sequences function as versatile templates that modulate metal nucleation and crystal growth kinetics (Fig. 2F and G). By exploiting specific coordination interactions between nucleobases and metal ions, these sequences regulate the size, composition, and exposed crystal facets of the resulting nanozymes [36]. This strategy allows for the rational synthesis of nanozyme heterostructures where the DNA sequence determines the catalytic performance through programmable base–metal interactions. Ultimately, this strategy improves synthetic controllability by reducing polydispersity and enabling more reproducible nanozyme fabrication.

### Interface and active site regulation

The catalytic efficacy of nanozymes is largely determined by their interfacial properties and the physicochemical characteristics of their active sites [6,37]. Interface and active site regulation primarily focuses on modulating catalytic kinetics, substrate accessibility, and interfacial interactions at the nanozyme surface. Interface properties influence substrate adsorption, accessibility, and interaction kinetics between nanozymes and their surrounding microenvironment [5,38], while the intrinsic features of active sites directly determine reaction turnover rates and selectivity [39]. In contrast to conventional physical encapsulation or chemical doping strategies, which often suffer from limited precision and controllability [37], DNA nanotechnology allows precise regulation of nanozymes interfaces and active sites [40]. Rather than serving merely as a scaffold, DNA in this mode functions as an interfacial regulator that reshapes substrate access, local charge environment, and catalytic microenvironment around nanozymes. This approach improves both catalytic stability and substrate specificity through precise control of catalytic interfaces.

#### Interface regulation

Interfaces define how nanozymes interact with their surrounding environment. DNA nanotechnology enhances catalytic performance by regulating 3 critical interfacial parameters: colloidal stability, substrate-binding affinity, and targeted recognition capabilities.

Maintaining colloidal stability under physiological conditions is essential for catalytic efficiency. In high-ionic-strength physiological environments, nanozymes are prone to nonspecific aggregation driven by electrostatic screening. This uncontrolled aggregation not only blocks active sites but also promotes rapid clearance by the reticuloendothelial system. To address this, DNA nanostructures provide steric stabilization, ensuring that nanozymes remain well dispersed during circulation until they reach their specific targets. For instance, Li et al. [41] functionalized Pt single-atom nanozymes (SAzymes) with rigid H-TDNs. This 3D protective layer inhibited particle aggregation, reducing the aggregation rate in phosphate-buffered saline (PBS) buffer from 45% to less than 8% and improving colloidal dispersion in serum by over 3-fold. Spectral analysis demonstrated that distinct DNA conformations exert differential effects on catalytic behaviors by regulating surface charge and steric accessibility [42,43].

DNA can regulate substrate affinity through sequence-dependent interactions and conformational modulation, as demonstrated by studies showing that DNA-mediated interfacial interactions enhance substrate enrichment near catalytic sites and improve binding kinetics [44]. Chen et al. [45] used DNA to direct the growth of platinum nanoparticles (PtNPs) on graphene oxide (GO) surfaces. Mechanistic analysis suggests that exposed amino and hydroxyl groups of DNA bases form hydrogen bonds with substrates [e.g., 3,3′,5,5′-tetramethylbenzidine (TMB)], thereby enriching local substrate concentration near the catalytic centers [46]. Kinetic analysis showed a 37% reduction in the Michaelis constant and a 2.5-fold increase in reaction rate. Consistent with these observations, Wang et al. [47] established a structure–function relationship regarding DNA conformation. Their work indicated that flexible ssDNA provides more contact sites than rigid double-stranded DNA (dsDNA), lowering the interfacial binding energy from −10.8 to −12.6 kcal mol^−1^. This thermodynamic advantage facilitates faster substrate adsorption and product desorption, enhancing peroxidase-like activity by 2.3-fold.

**Fig. 3.**
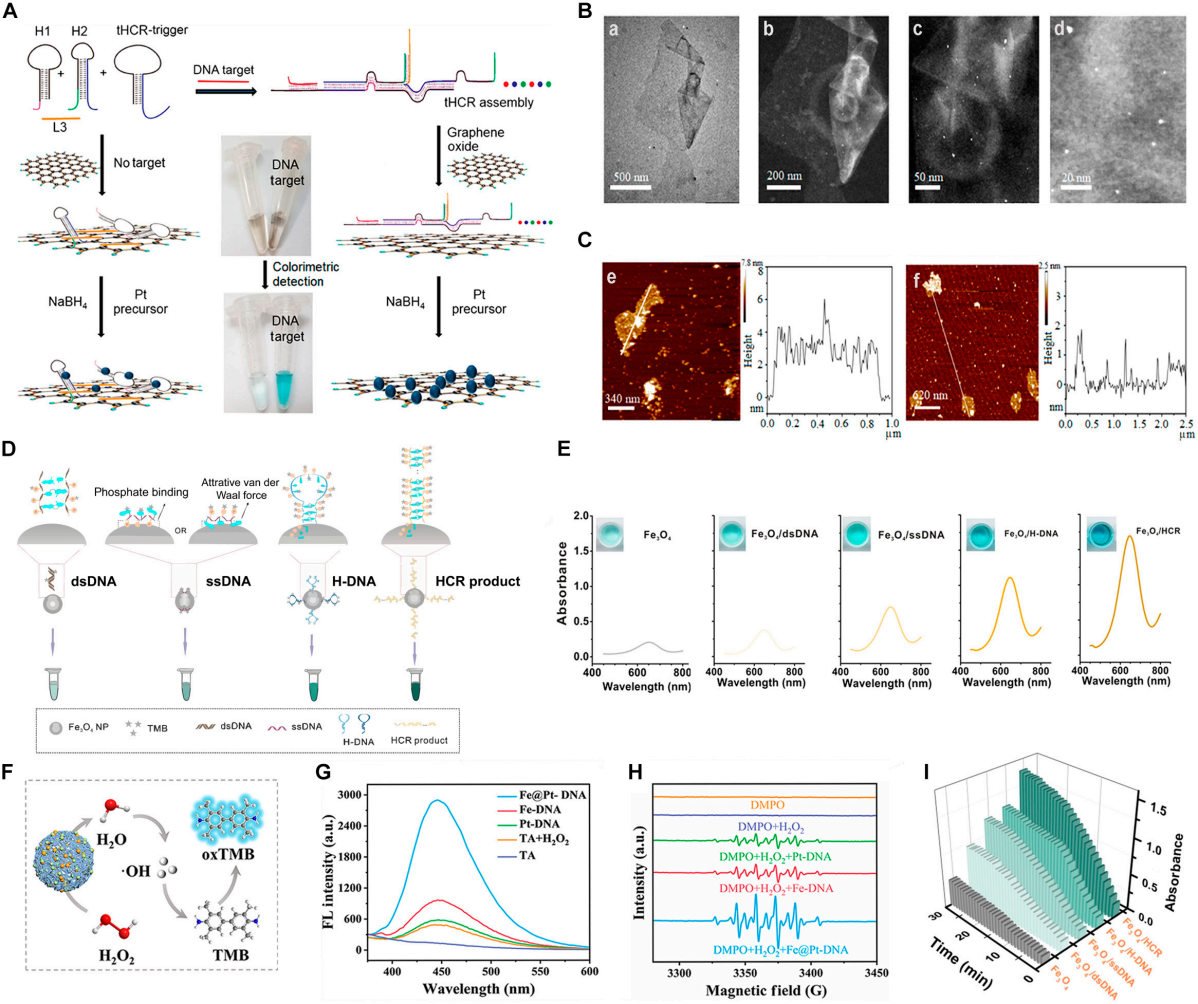
DNA nanotechnology-mediated interface/active site regulation of nanozyme activity. (A) Colorimetric nucleic acid detection strategy based on tHCR and DNA-controlled PtNP growth on GO. (B) TEM and STEM (scanning transmission electron microscope) images of GO/DNA-PtNPs. (C) AFM characterization of GO-PtNPs and DNA/GO-PtNPs. (A to C) Reproduced with permission from Ref. [45]. Copyright 2020 American Chemical Society. (D) Interaction between Fe_3_O_4_ nanoparticles and different DNA structures. (E) UV-vis spectra of TMB oxidation catalyzed by DNA-modified Fe_3_O_4_. (D and E) Reproduced with permission from Ref. [42]. Copyright 2018 American Chemical Society. (F) Fluorescence detection of ·OH using TA probe. (G) EPR spectra of ·OH radicals with DMPO spin trapping. (H) Proposed peroxidase-like catalytic mechanism of Fe@Pt-DNA. (F to H) Reproduced from Ref. [49]. Copyright 2024 Elsevier B.V. All rights reserved. (I) Time-dependent absorbance at 652 nm. Reproduced with permission from Ref. [42]. Copyright 2018 American Chemical Society.

The programmability of DNA hybridization also enables specific molecular recognition at nanozyme interfaces. Chen et al. [45] developed a dynamic interface based on a triple hybridization chain reaction (tHCR) for the in situ assembly of DNA structures on PtNPs@GO surfaces (Fig. 3A). Upon target recognition, hybridization initiates the assembly of long dsDNA probes, as visualized by transmission electron microscopy (TEM) and atomic force microscopy (AFM) (Fig. 3B and C). This dynamic functionalization strategy reduced nontarget interference from 35% to 5% and improved target recognition efficiency by 10-fold, enabling picomolar-level detection. Compared to static modification, this target-triggered assembly improves specificity and signal-to-noise ratios. AFM imaging and corresponding height profile analysis further confirmed the structural characteristics of the assembled DNA interface (Fig. 3D and E). While interface regulation mainly affects substrate approach and interfacial microenvironment outside the catalytic center, active site regulation operates more directly on the structure or chemical environment of the catalytic center itself.

#### Active site regulation

Interface engineering mainly influences substrate accessibility and interaction dynamics, whereas active site regulation determines catalytic turnover and reaction selectivity. The active site, typically composed of surface metal atoms, lattice defects, or functional groups, is responsible for substrate activation [48]. DNA nanotechnology enables the atomic-level modulation of active sites by regulating their density, electronic structure, and steric selectivity through metal-base coordination, π–π stacking, and electrostatic interactions (Fig. 3F).

A higher density of exposed active sites generally improves catalytic turnover. The catalytic activity was enhanced by approximately 4.7-fold compared with conventional synthesis methods. Conventional synthesis often results in site masking due to uncontrolled metal aggregation. DNA bases, acting as specific ligands, guide the oriented distribution of metal precursors. Zhang et al. [49] used the strong coordination affinity of DNA bases to direct the spatially confined co-reduction of Fe@Pt bimetallic nanozymes. This strategy increased active site exposure from 35% to 68% compared to traditional coprecipitation, resulting in a 4.7-fold enhancement in peroxidase-like activity (Fig. 3G). Electron paramagnetic resonance (EPR) spectroscopy indicated increased hydroxyl radical generation (Fig. 3H). Furthermore, optimizing the Fe/Pt stoichiometry (1:1.5) strengthened the electronic synergy between the metals, enabling a detection limit of 0.05 μM (Fig. 3I).

At the atomic level, DNA modulates the intrinsic electronic structure of catalytic centers. The electronic density and redox potential of active sites determine electron transfer efficiency [50]. Zhang et al. [51] suggested that interactions between DNA bases and metal centers can reshape the local electronic environment, facilitate charge transfer, and thereby enhance catalytic activity. Similarly, interactions between ssDNA and metal active sites may further optimize local redox cycling and contribute to improved catalytic performance.

Finally, substrate selectivity is achieved through steric gating and size-exclusion effects [52]. Confining nanozymes within DNA nanocages with defined pore sizes (e.g., 5 to 10 nm) creates a molecular sieve that permits the access of small-molecule substrates while excluding macromolecular interferents such as proteins. Notably, reversible DNA actuators, such as hairpins, enable the “on-demand” exposure of active sites, further minimizing nonspecific background catalysis in complex biological media [28,52].

### Precision delivery regulation

Although nanozymes show promising catalytic properties, their clinical translation is still limited by inefficient in vivo delivery. Following systemic administration, nanozymes often suffer from nonspecific biodistribution and rapid clearance by the mononuclear phagocyte system (MPS) [53]. Off-target accumulation reduces therapeutic efficacy and may increase toxicity due to uncontrolled reactive oxygen species (ROS) generation [54]. To reduce these challenges, DNA nanotechnology allows precise targeting and delivery systems through aptamer-mediated recognition, structure-dependent passive targeting, and synergistic conjugation with biological ligands [14,55].

#### Aptamer-mediated targeted delivery

Aptamers are single-stranded oligonucleotides selected through the systematic evolution of ligands by exponential enrichment (SELEX) process [47]. Due to their low molecular weight and programmable chemical modification, aptamers bind to target molecules with high affinity and specificity [19]. These properties make aptamers useful ligands for regulating the cellular uptake and intracellular trafficking of nanozymes.

**Fig. 4.**
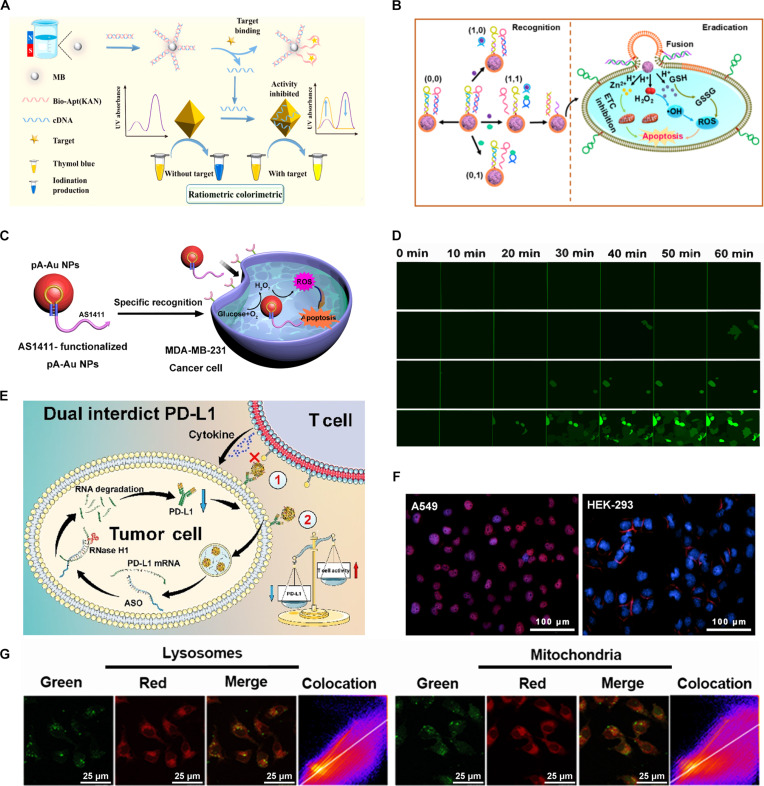
DNA nanotechnology-mediated precision delivery for regulating nanozyme activity. (A) Aptamer-regulated nanozyme-based ratiometric colorimetric detection. Reproduced from Ref. [35]. Copyright 2025 Elsevier B.V. All rights reserved. (B) DNA-based biosensing platform for cancer cell recognition and eradication. Reproduced with permission from Ref. [33]. Copyright 2025 American Chemical Society. (C) Targeted chemodynamic therapy of MDA-MB-231 cells using AS1411-functionalized pA-AuNP nanozymes. (D) Time-dependent ROS imaging in MCF-10A and MDA-MB-231 cells treated with pA-AuNPs or AS1411-pA-AuNPs. (C and D) Reproduced from Ref. [56] under the Creative Commons Attribution 4.0 International License (CC BY 4.0). (E) Dual PD-L1 blockade and gene silencing mediated by H-TDN. Reproduced from Ref. [41]. Copyright 2025 Elsevier Ltd. All rights reserved. (F) Cellular internalization in A549 and HEK-293 cells. Reproduced from Ref. [59] under the Creative Commons Attribution 4.0 International License (CC BY 4.0). (G) CLSM imaging and organelle colocalization in HeLa cells. Reproduced with permission from Ref. [33]. Copyright 2025 American Chemical Society.

Huang et al. [35] designed an aptamer-functionalized haloperoxidase-mimetic CeO_2_ nanozyme system, enabling ratiometric colorimetric sensing through aptamer-triggered conformational switching (Fig. 4A). In this design, the specific binding of the aptamer to its target induces the release of a complementary DNA strand, thereby modulating the catalytic activity of CeO_2_ for multicolor readout. Tang et al. [33] designed a logic-gated liposomal system responsive to high extracellular adenosine triphosphate (ATP) and specific membrane proteins (Fig. 4B). This dual-recognition mechanism ensures that membrane fusion is strictly confined to the tumor microenvironment, enabling the precise activation of nanozymes and demonstrating selective cytotoxicity in HeLa cell models.

Aptamer engineering can also support signal amplification and intracellular trafficking, improving the targeting precision and functional response of nanozyme systems. For instance, Han et al. [34] developed an aptasensor integrating ion-gated DNA nanorolls, where specific target binding drives a catalytic walking motor for cascade signal amplification. Similarly, aptamer-functionalized AuNPs were employed to construct cascade catalytic systems for selective tumor cell eradication [56]. For example, aptamer-functionalized nanomaterials can regulate nanozyme catalytic activity and enable target-responsive signal generation in biosensing systems [47,57]. Furthermore, subcellular targeting capabilities have been realized. DNA nanozymes functionalized with AS1411 aptamers (Fig. 4C) [58] achieve internalization via nucleolin-mediated endocytosis and subsequent nuclear translocation [59]. This mechanism allows nuclear delivery of photosensitizers, enhancing photodynamic therapy efficacy by over 3-fold. Time-dependent fluorescence imaging showed nuclear accumulation and in situ ROS generation (Fig. 4D) [59].

#### Size-mediated targeted delivery

DNA self-assembly allows precise control over nanostructure geometry and hydrodynamic diameter, which influences the in vivo pharmacokinetic profile and passive targeting efficiency [60]. DNA tetrahedra with dimensions of approximately 10 to 20 nm can escape renal filtration while avoiding rapid clearance by the reticuloendothelial system, thereby maximizing cellular uptake efficiency compared to linear DNA strands [14].

Current strategies use these structural properties to use the enhanced permeability and retention (EPR) effect observed in preclinical tumor models. Li et al. [41] designed Pt SAzymes modified with H-TDNs. Leveraging the mechanical rigidity and defined nanoscale dimensions (5 to 20 nm) of the DNA framework, these complexes exhibit prolonged circulation times and effective extravasation through leaky tumor vasculature. This passive enrichment provides a foundation for subsequent active targeting processes [55]. Moreover, the H-TDN scaffold simultaneously blocks the PD-1/PD-L1 pathway, achieving dual inhibition of immune checkpoint signaling to improve the antitumor immune response (Fig. 4E) [41]. This study shows how the structural parameters of DNA nanostructures determine multidimensional targeting and therapeutic efficacy.

Additionally, rolling circle amplification (RCA) technology facilitates the construction of larger DNA nanocomposites with high payload capacities [61]. Wang et al. [62] developed DNA nanocomposites (DNFs@ZnMn) incorporating deoxyribozymes and ZnO_2_-Mn nanozymes. By precisely tuning the template design, the hydrodynamic diameter of these composites was optimized to match the window for the EPR effect. Fluorescence imaging in tumor models confirmed that this size-dependent accumulation creates a high-concentration local environment, supporting tumor-specific responses and synergistic therapy (Fig. 4F).

#### Antibody-mediated targeted delivery

DNA strands can also function as linkers to integrate nanozymes with biological ligands such as antibodies, combining the programmability of DNA with the high specificity of antibody–antigen recognition. In such hybrid systems, antibodies guide the selective binding of nanozyme complexes to tumor-associated antigens [e.g., HER2 or epidermal growth factor receptor (EGFR)], while DNA scaffolds ensure the stable loading and spatial orientation of catalytic nanomaterials [24].

This strategy combines the high specificity of antibodies with the structural versatility of DNA, showing significant potential for precision tumor therapy [38]. Compared to single-ligand strategies, these DNA–antibody conjugates offer enhanced modularity. Subcellular colocalization analysis verified the targeted accumulation of these complexes in lysosomes and mitochondria (Fig. 4G). Compared with aptamer-based systems, antibody-mediated targeting generally provides higher binding affinity and well-established clinical translational potential.

DNA nanotechnology provides a platform for integrating multiple targeting modalities, enabling nanozyme delivery systems that combine tissue-specific accumulation, cellular recognition, and subcellular localization.

### Stimulus-responsive regulation

Precise spatiotemporal control of catalytic activity is important for nanozyme function in complex biological systems. DNA nanotechnology provides a platform for constructing stimulus-responsive nanozymes because of the programmability and reversible hybridization properties of DNA [63]. In these systems, DNA controls the accessibility of nanozyme active sites through structural transitions.

These mechanisms generally include chemically triggered responses, biomolecular recognition responses, and physicochemical stimuli such as light. In the absence of external stimuli, DNA generally inhibits catalytic activity through steric hindrance or active site blocking mediated by adsorption or coordination [64,65]. Upon exposure to specific triggers, DNA undergoes conformational changes or strand displacement. These structural transitions modulate the catalytic state between OFF and ON modes, which enables applications in biosensing, precision medicine, and asymmetric catalysis [66,67].

#### pH responsiveness

pH-responsive regulation often results from protonation of cytosine residues. This protonation destabilizes Watson–Crick base pairing and promotes the formation of noncanonical structures such as i-motifs [52]. In acidic microenvironments (pH 5.5 to 6.5), protonation induces conformational changes or structural disassembly of DNA–nanozyme complexes [68]. Conversely, under physiological conditions (pH 7.4), DNA maintains a stable conformation that suppresses catalytic activity [52].

**Fig. 5.**
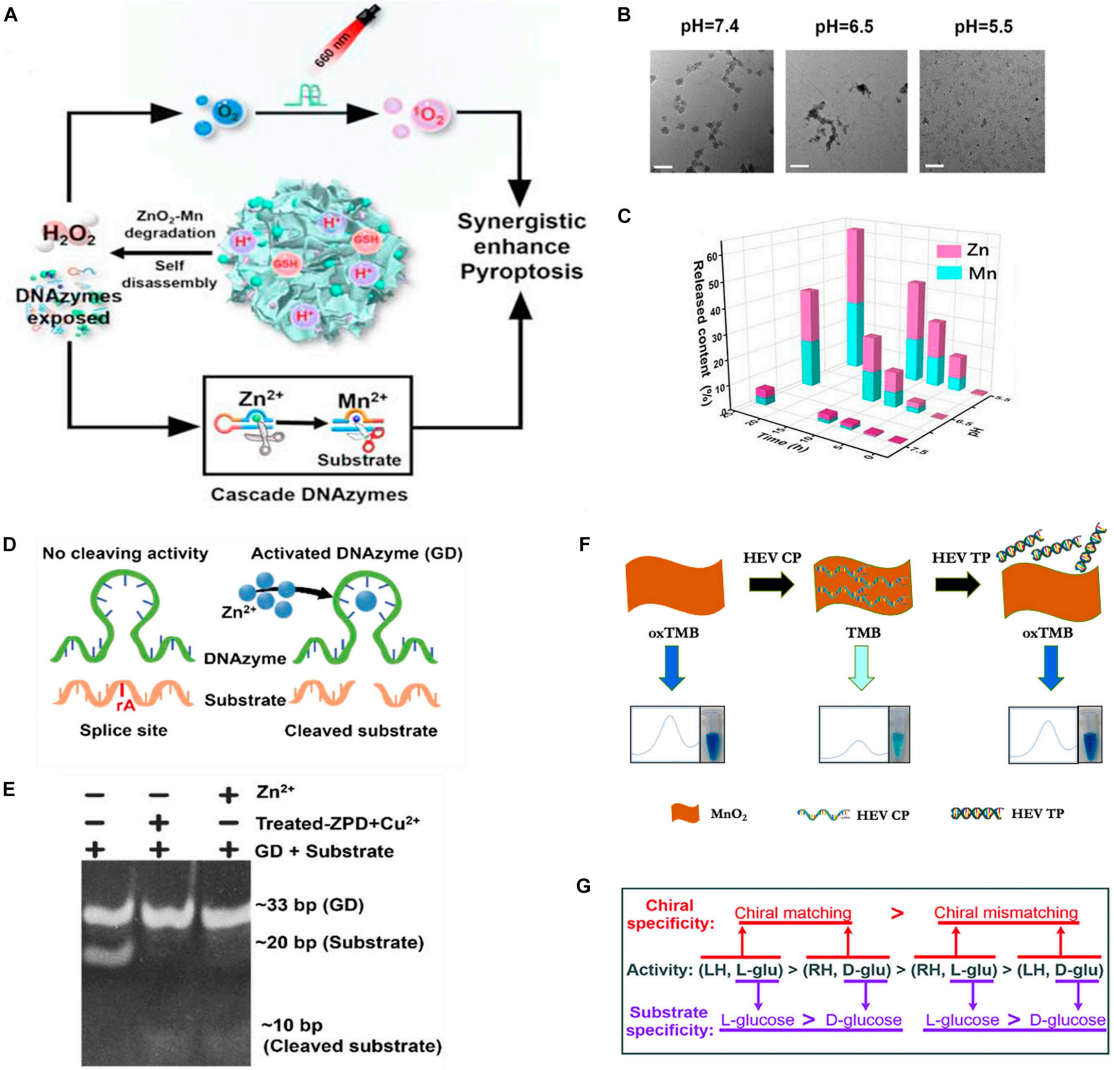
DNA nanotechnology-mediated stimulus-responsive regulation of nanozyme activity. (A) Schematic illustration of cascade catalytic reactions enabling synergistic mRNA cleavage to disrupt autophagy and alleviate tumor hypoxia. (B) TEM images of DNFs@ZnMn under different pH conditions (pH 7.4, 6.5, and 5.5). (C) pH-dependent release profiles of Zn^2+^ and Mn^2+^ from DNFs@ZnMn. (A to C) Reproduced from Ref. [62] under the Creative Commons Attribution 4.0 International License (CC BY 4.0). (D) Mechanism of DNAzyme-mediated mRNA cleavage. (E) Gel electrophoresis analysis of GD cleavage activity. (D and E) Reproduced from Ref. [73] under the Creative Commons Attribution 4.0 International License (CC BY 4.0). (F) MnO_2_-TMB nanozyme system for colorimetric detection of HEV. Reproduced with permission from Ref. [76]. Copyright 2022, Springer-Verlag GmbH Germany, part of Springer Nature. (G) Influence of substrate chirality and chiral matching on coronazyme catalytic activity. Reproduced from Ref. [84] under the Creative Commons Attribution 4.0 International License (CC BY 4.0).

For instance, Wang et al. [62] constructed a DNFs@ZnMn nanocomposite that undergoes acid-triggered decomposition within the tumor microenvironment. This process releases Zn^2+^ and Mn^2+^ cofactors, which subsequently activate DNAzyme-1 and DNAzyme-2 for the cascade cleavage of nucleic acid substrates (Fig. 5A). TEM confirmed this pH-dependent structural dissociation (Fig. 5B), while ion release profiles validated the cascade activation mechanism (Fig. 5C).

#### Metal ion responsiveness

Metal ion responsiveness relies on metal-base coordination interactions, particularly the formation of stable metallo-base pairs such as T-Hg^2+^-T and C-Ag^+^-C [69]. Metal ions provide vacant orbitals to coordinate with nucleobases, inducing conformational switching that modulates the interaction between DNA and the nanozyme surface [70].

Metal-ion-mediated base pairing can also regulate nanozyme activity by inducing conformational changes in DNA structures. For example, Hg^2+^ can specifically bridge thymine bases to form T-Hg^2+^-T mismatches. This interaction induces structural switching of DNA probes and modulates catalytic activity [71,72]. The specific binding of Hg^2+^ triggers the release of the inhibitory DNA strand, restoring peroxidase activity for highly sensitive Hg^2+^ detection [limit of detection (LOD): 10.5 nM]. Similarly, Zhou et al. [73] exploited a cation exchange reaction between ZnS nanoparticles and intracellular Cu^2+^. This process releases Zn^2+^, which acts as a cofactor to activate the GD DNAzyme for the targeted cleavage of Glut1 mRNA. The schematic of this ion-triggered activation is illustrated in Fig. 5D, with gel electrophoresis verifying the Zn^2+^-dependent cleavage efficiency (Fig. 5E).

#### Target nucleic acid response

Target nucleic acid regulation relies on toehold-mediated strand displacement and hybridization-induced conformational transitions. Specifically, the hybridization of target DNA or miRNA with a probe sequence induces a structural transition from single-stranded to double-stranded DNA [74]. This transition reduces the affinity of DNA for the nanozyme surface, which exposes previously blocked active sites [75].

For example, Alam et al. [76] adsorbed hepatitis E virus (HEV)-specific ssDNA onto MnO_2_ nanosheets to inhibit oxidase activity. In the presence of target DNA, hybridization results in the formation of rigid dsDNA, which detaches from the MnO_2_ surface due to electrostatic repulsion and steric hindrance (Fig. 5F). This restoration of activity enables femtomolar-level detection (3.26 fM). Furthermore, Broto et al. [69] integrated CRISPR-Cas13a collateral cleavage with nanozyme signal amplification to develop the CrisprZyme system. Upon recognizing target RNA (e.g., miR-223), the activated Cas13a cleaves reporter RNA strands [77]. This cleavage event prevents the immobilization of Pt@Au nanozymes on the assay plate, enabling amplification-free quantification of noncoding RNAs.

#### Target protein response

Protein-responsive regulation relies on the differential binding affinity between DNA aptamers and specific protein targets [78]. Upon target binding, the aptamer undergoes a conformational change that triggers its desorption from the nanozyme surface, creating an OFF–ON catalytic switch [79].

Protein adsorption on nanozyme surfaces can block access of substrates to catalytic sites and suppress catalytic activity. Target-induced desorption or structural switching of DNA probes can subsequently restore catalytic activity, thereby enabling selective biosensing [78,80]. The addition of a high-affinity bovine serum albumin (BSA) aptamer competitively strips the protein from the surface, re-exposing active sites. In a therapeutic context, Ouyang et al. [56] designed AS1411-modified pA-AuNPs targeting nucleolin. The specific binding of the AS1411 aptamer facilitates receptor-mediated endocytosis. Subsequent conformational changes activate the glucose oxidase-like activity of the nanozyme, generating H_2_O_2_ that is converted into toxic hydroxyl radicals. This target-activated cascade significantly enhances tumor-specific cytotoxicity compared to nontargeted controls [56,81].

#### Light responsiveness

Electron transport along chiral biomolecules such as DNA can exhibit spin selectivity [82]. This phenomenon, termed the chirally induced spin selectivity (CISS) effect, enables enantioselective catalysis [83].

Ji et al. [84] constructed a DNA-aptamer-modified Au nanoparticle system (coronazyme) where circularly polarized light (CPL) modulates catalytic efficiency (Fig. 5G). Mechanistic studies revealed that right-handed CPL selectively enhances catalytic efficiency toward d-glucose, whereas left-handed CPL promotes catalysis toward L-glucose. This polarization-dependent regulation achieved up to a 30-fold increase in catalytic efficiency.

These stimulus-responsive strategies show the capability of DNA nanotechnology to dynamically regulate nanozyme catalysis. By integrating molecular recognition, structural reconfiguration, and external stimuli responsiveness, these systems provide a powerful platform for constructing adaptive catalytic networks in complex biological environments.

### Cascade catalytic regulation

Cascade catalytic systems mimic multi-enzyme reaction pathways found in living organisms [6]. In contrast to static spatial organization, cascade catalytic regulation emphasizes dynamic substrate channeling and sequential catalytic coupling within spatially confined systems, enabling efficient intermediate transfer and reaction amplification. Cascade catalysis involves the sequential conversion of substrates through multiple catalytic steps within an integrated system. The fundamental principle involves utilizing DNA nanostructures as molecular scaffolds to organize the precise spatial assembly of heterogeneous catalytic units, including nanozymes, DNAzymes, and native enzymes [40,85]. Confined reaction pathways allow intermediates generated upstream to be rapidly transferred to downstream active sites. These architectures reduce diffusion limitations by enabling substrate channeling between adjacent catalytic units, thereby enhancing local intermediate concentration and overall catalytic efficiency.

DNA nanotechnology shows high programmability for this purpose, enabling precise control over the nanoscale spacing and orientation of catalytic units through complementary base pairing or specific chemical modifications [40,86]. Furthermore, DNA-engineered confined spaces, ranging from nanointerfaces to hydrogels, effectively minimize the dissipation of active intermediates and foster synergistic interactions between catalytic units [25,87]. Recent studies report nanozyme–nanozyme cascades and hybrid nanozyme-enzyme systems, showing strong potential in biosensing and targeted therapy [88].

#### Catalytic relay cascades

Catalytic relay cascades rely on the spatial organization to assemble multiple nanozymes into proximity-dependent catalytic systems [20,23]. This configuration enables substrate channeling, allowing intermediates generated upstream to reach downstream catalytic sites rapidly. By imposing spatial confinement, DNA scaffolds shorten the diffusion path of intermediates, which reduces activity loss and improves reaction kinetics [88].

For instance, Wang et al. [62] used DNA sequence specificity to design a dual-functional relay system. In this architecture, DNA serves as a structural scaffold for Zn^2+^-dependent DNAzyme-1 and Mn^2+^-dependent DNAzyme-2. At the same time, it also acts as a template for ZnO_2_-Mn nanocatalysts. In the acidic tumor microenvironment, the ZnO_2_-Mn core decomposes to release Zn^2+^ and Mn^2+^ cofactors, which sequentially activate both DNAzymes for mRNA cleavage. Concurrently, the H_2_O_2_ generated from ZnO_2_ decomposition is subsequently decomposed by MnO_2_ to produce O_2_, alleviating tumor hypoxia and inducing pyroptosis. Here, the dual role of DNA as both a scaffold and a substrate channel ensures the synchronization of cofactor release with catalytic propagation [62,63].

**Fig. 6.**
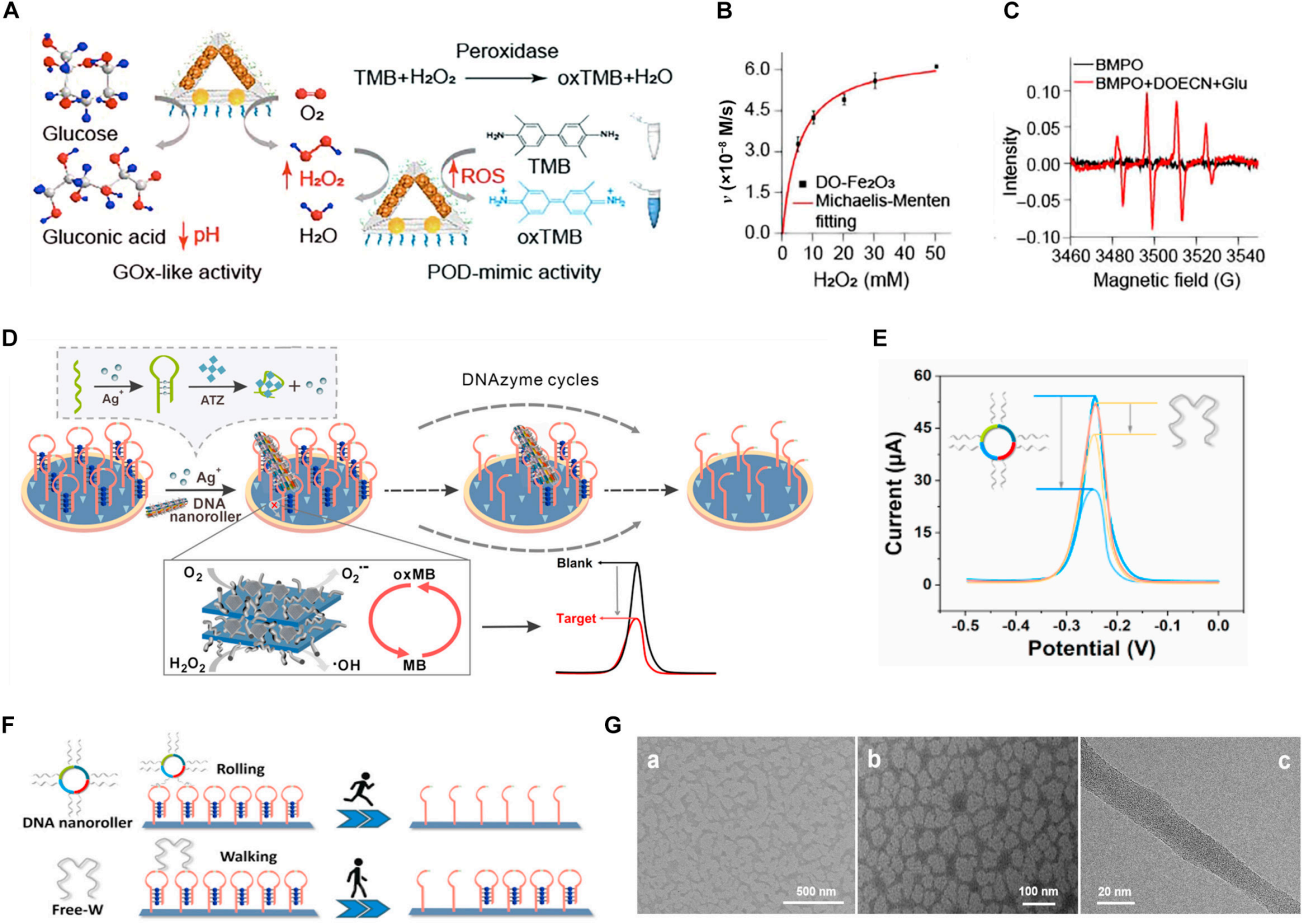
DNA nanotechnology-mediated cascade catalysis for regulating nanozyme activity. (A) Schematic illustration of the cascade reaction process of DNA origami-based enzymatic cascade nanoreactor (DOECN) with GOx-like and POD-like activity. (B) Michaelis–Menten kinetic analysis of DO-Fe_2_O_3_. (C) 5-Tert-butoxycarbonyl-5-methyl-1-pyrroline N-oxide (BMPO) spin-trapping ESR spectra of DOECN in the presence of glucose. (A to C) Reproduced from Ref. [26] under the Creative Commons Attribution 4.0 International License (CC BY 4.0). (D) Diagrammatic representation of the aptasensor detection procedure. (E) DPV curves and (F) schematic of operating with different nanoroller devices. (G) TEM images (a to c) of the DNA nanorollers. (D to G) Reproduced from Ref. [34] Copyright 2025 Elsevier B.V. All rights reserved.

Alternative strategies employ DNA to create spatially confined environments, enabling multiple catalytic activities to synergize on a single nanoparticle. Ji et al. [84] developed a DNA aptamer-modified AuNP system (coronazyme) where the helical DNA structure provides chirality to the AuNPs while spatially enriching glucose on the surface (Fig. 6A). Mechanistic studies revealed a 30-fold increase in reaction rate compared to unmodified controls, as confirmed by Michaelis–Menten kinetic analysis (Fig. 6B). In this system, AuNPs catalyze glucose oxidation to generate H_2_O_2_, which is confined within the DNA layer and immediately used by the peroxidase active site to oxidize the chromogenic substrate. This spatial confinement effectively creates a pseudo-cascade environment in which the intermediate H_2_O_2_ is locally accumulated and rapidly consumed. Electron spin resonance (ESR) analysis verified the efficient generation of ROS (Fig. 6C).

#### Nucleic acid amplification cascades

Nucleic acid amplification cascades combine isothermal amplification techniques with nanocatalyst-driven reactions to convert target recognition events into detectable outputs [89,90]. Typically, target binding triggers an amplification process, such as the hybridization chain reaction (HCR) or RCA [91–93]. These dynamic processes generate abundant DNA products that serve as scaffolds to recruit high-density nanozymes or enrich substrates, forming a recognition–amplification–signal transduction cascade [90].

Han et al. [34] developed an electrochemical sensing platform for atrazine detection, where target-triggered dissociation of C–Ag^+^–C mismatches activates a DNA nanoroller motor (Fig. 6D). Target recognition releases Ag^+^ ions, which activate the DNA nanoroller motor to continuously cleave hairpin probes. This cycle generates a high concentration of methylene blue signal molecules. Concurrently, nanocatalysts immobilized at the electrode interface efficiently catalyze the oxidation of these molecules, yielding a highly sensitive electrochemical response. Differential pulse voltammetry confirmed the signal amplification (Fig. 6E). By integrating the roles of recognition probe, scaffold, and immobilization platform, this system achieved a detection limit of 2 × 10^−5^ ng/ml [34]. The nanoroller-driven amplification process and the corresponding catalytic reaction scheme are illustrated in Fig. 6F.

DNA nanotechnology also enables the construction of programmable catalytic networks through dynamic assembly reactions. Programmable DNA assembly processes, such as HCR, can generate extended nucleic acid scaffolds that spatially organize nanozymes and enhance catalytic efficiency, thereby providing effective platforms for signal amplification and biosensing applications (Fig. 6G) [42]. In this system, the DNA amplification products function simultaneously as structural scaffolds, catalytic activity enhancers, and substrate enrichment carriers, thereby maximizing signal output [93].

Collectively, DNA-mediated cascade catalytic systems provide a powerful strategy to integrate multiple catalytic processes within spatially organized nanostructures. By combining substrate channeling with nucleic acid amplification, these architectures significantly enhance catalytic efficiency and signal sensitivity, offering broad potential for biosensing and precision therapeutics.

### Stability regulation

**Fig. 7.**
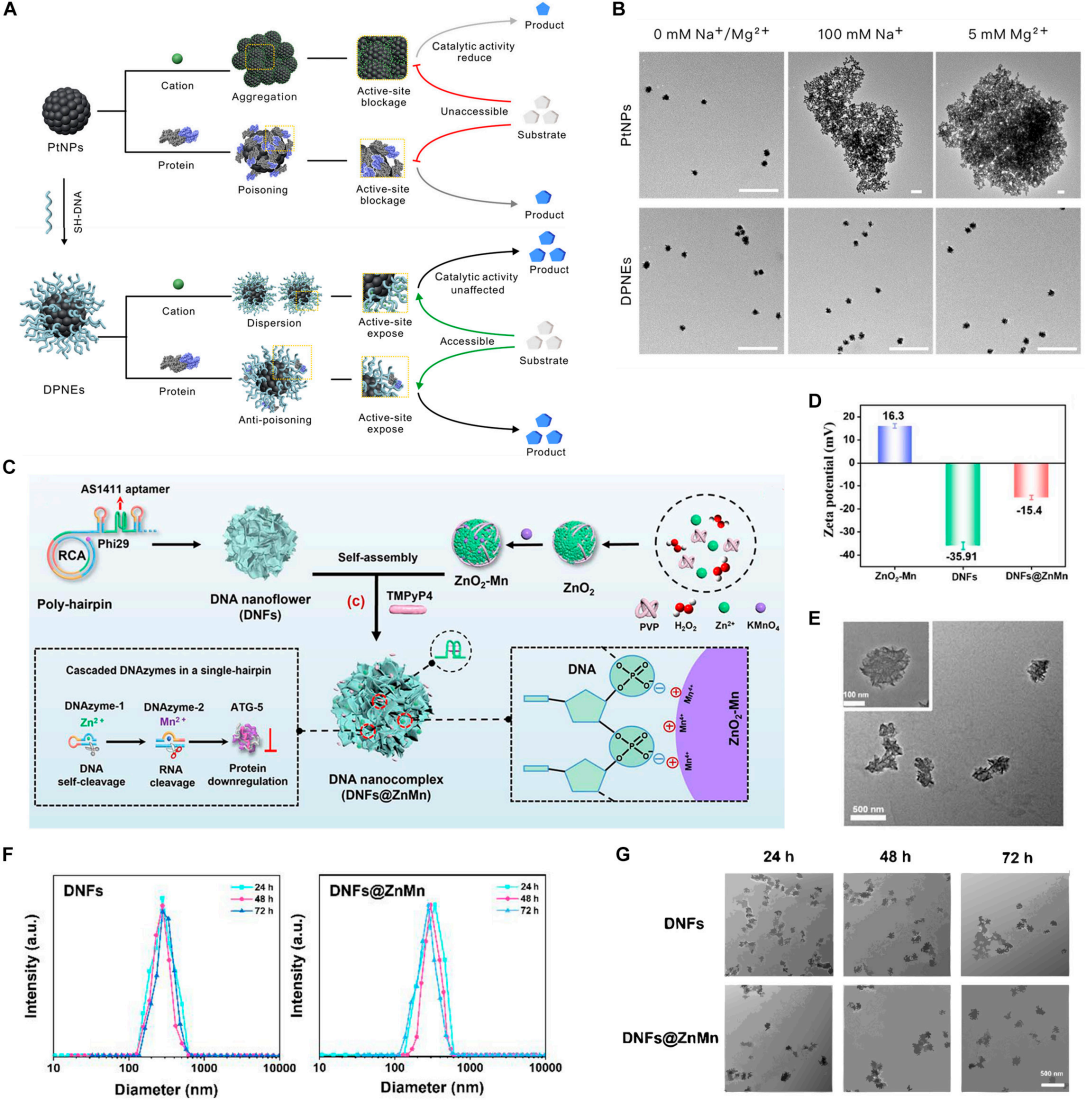
DNA nanotechnology-mediated stabilization strategies for nanozyme activity. (A) DNA-engineered coatings protecting Pt nanozymes from aggregation and protein poisoning. (B) TEM images of PtNPs and DNA-protected nanozymes under different ionic conditions. (A and B) Reproduced from Ref. [94] under the Creative Commons Attribution 4.0 International License (CC BY 4.0). (C) Assembly of DNFs@ZnMn based on RCA-generated DNA nanoflowers and ZnO_2_-Mn loading. (D) Zeta potential characterization of ZnO_2_-Mn, DNFs, and DNFs@ZnMn. (E) TEM images of DNFs@ZnMn. (F) Dynamic light scattering (DLS) analysis in PBS buffer. (G) Time-dependent TEM images showing structural stability. (C to G) Reproduced from Ref. [62] under the Creative Commons Attribution 4.0 International License (CC BY 4.0).

The application of nanozymes is often limited by their susceptibility to environmental stressors, including biofouling, aggregation, and oxidative degradation [94]. In contrast, stability regulation is primarily concerned with preserving the structural integrity and catalytic functionality of nanozymes under complex environmental conditions. DNA-mediated stability regulation involves constructing protective shielding systems that preserve nanozyme performance in complex biological and environmental conditions. As schematically illustrated in Fig. 7A, DNA scaffolds act as steric and electrostatic protection layers that counteract serum protein interference and high-salt erosion. This strategy maintains structural integrity and catalytic activity without blocking active sites or altering intrinsic kinetic parameters [94]. DNA-mediated stabilization improves nanozyme performance in complex biological and environmental matrices [94,95]. This protective effect is directly evidenced by TEM images (Fig. 7B), which show that DNA-engineered nanozymes remain well dispersed under ionic stress, whereas unmodified counterparts undergo pronounced aggregation.

#### Stability against serum protein interference

In complex biological fluids, the formation of a nonspecific protein corona can block active sites and diminish catalytic efficiency [8]. DNA coatings with high hydrophilicity and negative charge density create a dense hydration layer that blocks nonspecific protein adsorption [47,96].

Wang et al. [62] used DNA nanoblossoms to encapsulate ZnO_2_-Mn nanozymes. Beyond preventing protein fouling, the hierarchical assembly of DNA nanoflowers (schematically presented in Fig. 7C) provides robust colloidal stability. Surface charge analysis confirmed the successful DNA modification, as reflected by the shift in zeta potential values (Fig. 7D). The morphology of the assembled DNFs@ZnMn complex was further confirmed by TEM imaging (Fig. 7E), which revealed the formation of compact nanostructures after nanozyme loading. After incubation in 10% fetal bovine serum for 24 h, the DNA-protected system retained 92% of its activity with a Michaelis constant for H_2_O_2_ (0.87 mM) nearly identical to that of the pristine state (0.85 mM). In contrast, the unmodified group retained only 58% activity. Similarly, in the polyA-modified AuNP system developed by Ouyang et al. [56], the DNA corona enabled ≥90% activity retention after incubation in serum for 7 d, whereas the unmodified group exhibited a 50% activity decline within 3 d. These results indicate that DNA modification effectively preserves the intrinsic catalytic kinetics of both glucose oxidase (GOx)-like and peroxidase (POD)-like activities by preventing surface fouling.

#### Resistance to enzymatic degradation

In addition to passive fouling, DNA–nanozyme hybrids need to resist enzymatic cleavage by serum nucleases, ensuring functional persistence [97]. Covalent conjugation (e.g., Au–S bonds) or dense 3D encapsulation shields the DNA–nanozyme interface from nuclease attack.

Mei et al. [98] demonstrated that DNA strands anchored onto Pt nanozymes through strong metal–thiol interactions enhance structural stability in nuclease-rich environments. After incubation in deoxyribonuclease (DNase)-containing serum, the DNA-modified nanozyme system maintained its peroxidase-like catalytic activity, indicating effective protection of the catalytic interface. Similarly, Li et al. [28] reported that DNR-encapsulated copper nanoclusters (Cu NCs) retained 88% of their SOD/catalase (CAT)-like activity after exposure to DNase I for 12 h, with unchanged substrate affinity.

#### Stability under high-salt conditions

High ionic strength environments induce irreversible aggregation of nanomaterials due to the screening of surface charges [95]. DNA scaffolds reduce this effect by providing strong electrostatic repulsion and steric stabilization, thereby maintaining monodispersity [49,94].

TEM reveals the preservation of dispersion for DNA-modified systems under high-salt conditions, in sharp contrast to the aggregation observed in unmodified controls. DNA modification prevents salt-induced aggregation of nanozymes by providing electrostatic repulsion and steric stabilization, enabling nanozymes to maintain colloidal stability and catalytic activity even under high-ionic-strength conditions such as concentrated NaCl solutions [6]. Conversely, unmodified nanozymes underwent significant agglomeration, with particle sizes increasing from 32 nm to 210 nm. These results indicate that DNA scaffolds provide an effective barrier against salt-induced aggregation. It should be noted that quantitative comparisons across different nanozyme systems remain challenging due to variations in experimental conditions and reporting standards in the literature.

#### Catalytic durability

Operational durability, including recycling capability and long-term storage stability, is critical for industrial viability [99]. DNA modification restricts conformational fluctuations and protects nanozymes from environmental oxidants, thereby enhancing robustness [37].

Regarding recycling stability, the robust anchoring of DNA minimizes active site leaching during catalysis. Lapshinov et al. [96] reported that platinum nanozymes modified with T10 sequences retained over 80% peroxidase-like activity after 5 freeze–thaw cycles. Similarly, Yang et al. [19] observed that thiolated ssDNA-coated nanozymes maintained 85% activity after 5 catalytic cycles, significantly outperforming the unmodified control (40%). Dynamic light scattering analysis (Fig. 7F) and time-dependent TEM observations (Fig. 7G) further verified the long-term structural integrity of these systems. In terms of storage, DNA coatings create an inert microenvironment that shields active sites from oxidation. Lapshinov et al. [96] showed that T10-modified nanozymes stored at 4 °C for 10 d exhibited no significant decline in catalytic rate constants. Li et al. [28] further demonstrated that DNR-encapsulated systems retained over 90% activity after storage for 14 d. Because the cited studies employed different nanozyme compositions, substrates, assay conditions, and readout criteria, the reported quantitative metrics should be interpreted as representative performance indicators rather than directly interchangeable benchmarks. Additionally, a comparative summary of representative DNA-regulated nanozyme systems is provided in Table 2 to highlight their distinct regulatory modes, key performance metrics, and underlying design strategies.

**Table 2.** Representative DNA-regulated nanozyme systems and their key performance metrics

Regulatory mode	Representative system	Metric type	Quantitative performance	Reference
Spatial positioning regulation	DNA nanoribbon-templated Cu NCs	Activity	Significantly enhanced antioxidant activity	[25]
	ssDNA-templated bimetallic nanozyme	Catalytic efficiency	Higher activity than monometallic counterparts	[26]
Interface/active site regulation	H-TDN stabilized Pt single-atom nanozyme	Stability	Reduced aggregation; improved serum dispersion	[38]
	DNA-directed PtNPs on GO	Kinetics	K_m_ reduced; reaction rate increased	[42]
	DNA-coordinated Fe@Pt nanozyme	Activity	Active site exposure increased from 35% to 68%	[46]
Precision delivery regulation	Aptamer-gated liposomal nanozyme	Targeting	Tumor-specific activation with selective cytotoxicity	[30]
	DNA tetrahedral nanozyme system	Cellular uptake	Enhanced cellular internalization and tumor accumulation	[38]
Stimulus-responsive regulation	DNFs@ZnMn system	Activation	Acid-triggered ion release enabling cascade activation	[59]
	MnO_2_ nanosheet system	Sensitivity (LOD)	LOD = 3.26 fM	[73]
	CPL-regulated coronazyme	Activity	~30-fold activity difference under polarized light	[81]
Cascade catalytic regulation	DNA nanoroller electrochemical system	Sensitivity	LOD = 2 × 10^−5^ ng/ml	[31]
	DNA-confined cascade nanozyme system	Activity	~30-fold reaction rate increase due to substrate channeling	[59]
Stability regulation	DNA nanoflower-encapsulated nanozyme	Activity retention	~92% activity retained after 24 h in serum	[59]
	PolyA-modified nanozyme	Stability	≥90% retained after 7 d	[53]

Taken together, these 6 modes show that DNA does not regulate nanozymes through a single pathway, but through multiple structurally encoded mechanisms that affect catalytic efficiency, accessibility, activation timing, signal amplification, and operational robustness. On this basis, the following section discusses how these mechanistic advantages are translated into representative biomedical and analytical applications.

## Applications of DNA-Regulated Nanozymes

**Fig. 8.**
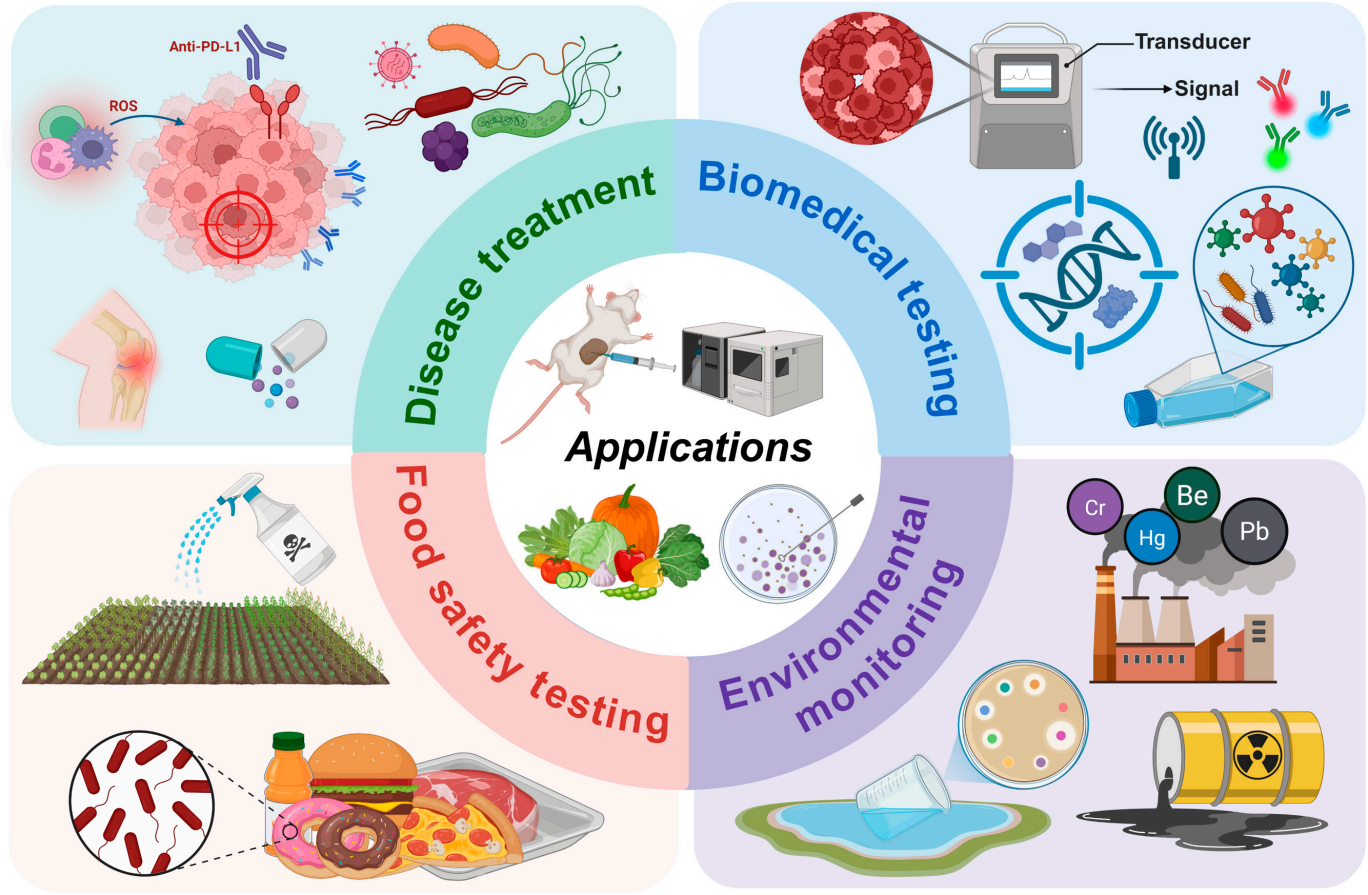
Versatile applications of DNA nanotechnology-engineered nanozymes: disease treatment, biomedical testing, food safety, and environmental monitoring. Created with BioRender.com.

The precise regulation of nanozymes via the 6 core strategies outlined above addresses intrinsic bottlenecks regarding stability and specificity, thereby broadening their practical applications in disease therapy, biomedical detection, environmental monitoring, and food safety monitoring. A comprehensive overview of these applications is illustrated in Fig. 8. Herein, we systematically analyze the core application scenarios and technical advantages of DNA-regulated nanozymes through representative research cases.

### Disease treatment

In therapeutic settings, DNA regulation enhances nanozyme function primarily through targeted accumulation, conditional activation within diseased microenvironments, and integration with other therapeutic modules such as photodynamic, ferroptotic, or immunomodulatory pathways [100–102]. These strategies have been applied in tumor therapy and antimicrobial treatments.

Tumor therapy is a major research direction for DNA-regulated nanozymes. Many strategies use the acidic or hypoxic tumor microenvironment to trigger nanozyme activity, thus maximizing therapeutic efficacy while minimizing off-target toxicity [103]. For instance, Wang et al. [62] constructed dynamic cascade DNA nanocomplexes (DNFs@ZnMn). The DNFs@ZnMn system represents a typical example integrating stimulus-responsive regulation and cascade catalytic regulation, together with stability-related advantages derived from the DNA nanoflower framework, which release active components in response to intratumoral acidity. These components disrupt autophagic flux through a DNase-mediated cascade targeting ATG5 while simultaneously producing O_2_ through ZnO_2_ decomposition to alleviate tumor hypoxia, thereby enhancing photodynamic therapy and reversing drug resistance. Similarly, Cao et al. [104] developed adaptive SAzymes, featuring surface-modified DNA regulators that specifically boost ROS production and deplete intracellular glutathione in tumor cells [73]. This mechanism selectively triggers ferroptosis and shows therapeutic effects in colon and breast cancer models.

Beyond direct catalytic tumor killing, DNA-regulated nanozymes function as immunomodulators to improve antitumor immunity [105]. Li et al. [41] designed Pt SAzymes modified with H-TDNs. In this system, the nanozyme alleviates hypoxia by generating O_2_, while the H-TDN moiety inhibits PD-L1 expression, thus enhancing immune checkpoint blockade efficacy and promoting T cell infiltration. Qiao et al. [106] reported a single-atom manganese nanozyme (Mn-N/C) that catalyzes Fenton-like reactions to induce immunogenic cell death. This process activates the cGAS-STING pathway to stimulate type I interferon production, improving the therapeutic effect of anti-PD-L1 therapy. These studies indicate that DNA-mediated regulation enables spatiotemporal control over nanozyme catalytic activity within tumor microenvironments, thereby improving therapeutic selectivity and enabling synergistic therapeutic modalities including photodynamic therapy, ferroptosis, and immunotherapy [107,108].

In addition to cancer therapy, DNA-regulated nanozymes have also been explored in antimicrobial applications, particularly for combating antimicrobial resistance (AMR) through stimulus-responsive catalytic activation. Chen et al. [109] developed ATP-activated cascade reactors (GGPcs) where catalytic activity depends strictly on bacterial ATP secretion. This ensures on-demand activation at infection sites. Additionally, Mo et al. [110] constructed a multi-enzyme platform (a SOD/Au@NiCoCu MOF-based antibacterial platform with dual enzyme-like activities) combining SOD-like and deoxygenase-like activities. This system converts H_2_O_2_ into highly reactive hydroxyl radicals (·OH) for bactericidal activity while simultaneously depleting intracellular glutathione to reduce ROS scavenging, demonstrating potent efficacy in treating bacterial wound infections.

### Biomedical detection

Biomedical detection relies on the precise identification and quantitative analysis of diverse molecular targets—including nucleic acids, proteins, small metabolites, and pathogens—within complex biological matrices [111,112]. DNA nanotechnology empowers nanozymes with high-precision recognition and catalytic signal amplification, enabling ultrasensitive detection across diverse biological matrices [113].

Among these targets, nucleic acid biomarkers represent one of the most widely investigated detection categories. DNA provides specificity via base pairing while nanozymes drive signal output [114]. Chen et al. [45] constructed a colorimetric sensor based on tHCR and Pt nanozymes, achieving a LOD of 14.6 pM for let-7a miRNA with single-nucleotide discrimination capabilities. To further enhance sensitivity, Gao et al. [115] integrated rolling hoop orbital amplification (RHOA) with cascade nanozyme catalysis, attaining an ultra-low LOD of 1.5 aM for exosomal miRNA-15a-5p. Alternatively, Alam et al. [76] used MnO_2_ nanosheets where hybridization-induced DNA dissociation restores nanozyme activity, enabling femtomolar detection sensitivity. More recently, Broto et al. [69] integrated the CRISPR/Cas13a system with nanozymes to establish the CRISPRZyme platform, which achieves amplification-free quantification of noncoding RNAs for acute myocardial infarction diagnosis.

Beyond nucleic acids, proteins can also be detected through DNA-regulated nanozyme platforms. For protein targets, aptamer binding induces conformational changes that modulate the nanozyme microenvironment [78,79]. Ali and Omer [116] employed a copper-based metal-organic framework (Cu-MOF) where thrombin binding restores fluorescence and catalytic activity. This mechanism allows dual-mode detection of coagulation markers in COVID-19 patients.

In addition to macromolecular biomarkers, DNA-regulated nanozyme platforms can also be applied to the detection of small-molecule targets. Small-molecule detection commonly relies on aptamer-mediated competitive binding or interface regulation [117]. Zou et al. [87] developed a sensor using hydrogel-encapsulated Au_0.4_Pt_0.6_ nanozymes. The binding of zearalenone (ZEN) triggers the dissociation of the aptamer–cDNA duplex, leading to hydrogel collapse and nanozyme release. This sensor achieves an LOD of 0.6979 ng/ml for ZEN.

DNA-regulated nanozyme systems have also been applied to pathogen detection. Pathogen detection often employs surface-modified aptamers to regulate activity via steric hindrance. Hu et al. [118] constructed a yolk-shell nanozyme sensor where the specific binding of aptamers to *Salmonella typhimurium* relieves steric hindrance. This structural dissociation restores catalytic activity for quantitative detection.

In the field of virology, Li et al. [119] developed a symmetric homotrimeric aptamer (TMSA52) that shows high-affinity molecular recognition toward the homotrimeric spike protein of severe acute respiratory syndrome coronavirus 2 (SARS-CoV-2). The trimeric architecture of TMSA52 enables multivalent interactions with the spike protein, significantly enhancing binding stability and specificity compared with conventional monomeric aptamers. This aptamer can recognize multiple SARS-CoV-2 variants and even pseudotyped viral particles, demonstrating its potential as a robust molecular recognition element for virus detection and antiviral diagnostics [119].

At the cellular level, DNA-regulated nanozyme biosensors have also been developed for cancer cell detection. Zhu et al. [39] developed a sandwich biosensor using aptamer-modified magnetic particles for cancer cell capture. Bimetallic MOF-based nanozymes bind directly to the cell surface, simplifying sensor design while achieving signal amplification.

### Environmental detection

DNA-regulated nanozyme sensors provide portable and cost-effective platforms for environmental monitoring, where specific molecular recognition events are translated into measurable catalytic signals [99].

One major application involves heavy metal ion detection, which typically relies on ion-induced conformational switching of DNA structures that regulate nanozyme catalytic activity through metal-base coordination interactions. For example, Mao et al. [120] engineered a paper-based analytical device modified with DNA-AuNPs. In this system, the specific binding of Hg^2+^ induces a conformational change in the DNA probe, triggering catalytic color development for on-site water quality analysis.

Radionuclide monitoring represents another important environmental application. In such systems, specific aptamers are often employed to reversibly regulate nanozyme catalytic activity. Zhang et al. [51] used a UO_2_^2+^-specific aptamer to regulate the catalytic activity of Tph-BT nanozymes. Upon uranium binding, the aptamer desorbs from the nanozyme surface, restoring catalytic activity and enabling the multicolor colorimetric detection of uranium ions.

In addition, DNA-regulated nanozyme systems have been applied to antibiotic contamination monitoring. Integrated platforms combining CRISPR or Cas systems with magnetic nanozymes have recently been developed**.** Zhang et al. [121] reported a CRISPR-driven tri-mode nanozyme platform for the detection of ampicillin and β-lactam resistance genes. By targeting specific resistance genes, these platforms integrate CRISPR-mediated recognition with efficient nanozyme catalysis, enabling ultrasensitive detection in complex environmental matrices such as water and soil.

### Food safety monitoring

Food safety monitoring requires the rapid identification of biological and chemical contaminants using portable and integrated analytical platforms suitable for on-site screening [47].

To facilitate on-site detection of pesticide residues, DNA-regulated nanozymes are frequently encapsulated within functional matrices. Zou et al. [122] synthesized Fe–G nanozymes encapsulated in alginate aerogels, forming a porous scaffold that enhances substrate diffusion and catalytic efficiency for the smartphone-based detection of chlorpyrifos. Extending this strategy to mycotoxin monitoring, Sun et al. [123] constructed an aptamer-responsive HA-DNA hydrogel integrated with a bimetallic MOF nanozyme for the sensitive colorimetric detection of ZEN in grains, where target binding induces hydrogel disintegration and activates catalytic signal generation.

For biological contaminants, DNA-regulated nanozyme platforms have also demonstrated strong potential. Dual-mode platforms and steric regulation strategies are employed to enhance accuracy. Ma et al. [124] established a fluorescence/colorimetric dual-mode platform for identifying aflatoxin B1 in peanuts and milk powder, using DNA to strictly control peroxidase-like activity. For bacterial pathogens, Hu et al. [118] designed an aptamer-regulated eggshell nanocatalyst sensor for *S. typhimurium*, where the aptamer functions as a reversible gatekeeper to modulate active site accessibility. Collectively, these studies highlight the potential of DNA-regulated nanozyme platforms as robust analytical tools for the rapid on-site screening of food contaminants. DNA-regulated nanozyme sensors offer portable and cost-effective solutions for food safety monitoring due to their stability and rapid response.

## Conclusions and Outlook

DNA nanotechnology provides an effective approach to address current limitations in nanozyme specificity and catalytic efficiency owing to its programmable nature, molecular recognition capability, and biocompatibility. By linking biomolecules and inorganic nanomaterials, this field has expanded the design possibilities of bio-orthogonal catalysis. This review summarizes recent advances in DNA-mediated nanozyme regulation and highlights the key regulatory principles that control these systems. In most cases, they together affect spatial organization, interfacial interactions, and dynamic responsiveness, which shape the catalytic microenvironment and ultimately determine overall activity. Despite this progress, several issues still limit the practical application of DNA-regulated nanozymes from laboratory studies to clinical and industrial applications. Future work should address these challenges through the following 5 key directions. This review also discusses how these regulatory strategies have been applied in disease theranostics, biomedical diagnostics, environmental monitoring, and food safety testing, highlighting the importance of this interdisciplinary field.

### Scalable production and cost reduction

The large-scale production of DNA-regulated nanozymes remains a major challenge. The construction of complex DNA nanostructures, such as DNA origami or multidimensional FNAs, still relies on the precise assembly of numerous high-purity oligonucleotides, which significantly increases manufacturing costs and limits practical translation. In addition, the self-assembly process is highly sensitive to thermodynamic conditions, resulting in batch-to-batch variability in structural uniformity and catalytic performance.

Developing scalable and automated manufacturing strategies will be essential for practical translation. Enzymatic synthesis approaches (e.g., RCA) and microfluidic-assisted assembly may enable cost-effective production and better control of assembly conditions. In addition, biomanufacturing platforms based on engineered microorganisms or cell-free systems may offer new routes for large-scale DNA scaffold production.

### Enhancing in vivo stability and biosafety

Physiological stability remains a major limitation for DNA-based nanozyme systems. In complex biological environments, DNA nanostructures are susceptible to nuclease degradation and rapid clearance by the reticuloendothelial system, which shortens their circulation time and weakens regulatory effects in vivo. Furthermore, systematic studies on long-term biosafety, immunogenicity, and metabolic pathways remain insufficient.

Future research should focus on the development of more robust architectures through nuclease-resistant nucleic acid analogues, surface shielding strategies, and optimized structural designs. Comprehensive biosafety evaluation and pharmacokinetic studies will also be essential for advancing clinical translation.

### Interference resistance in complex systems

In practical applications, DNA-based nanozyme systems often encounter interference from complex biological or environmental matrices. Protein adsorption, nonspecific ion binding, and biomolecular fouling may induce structural aggregation or block catalytic active sites, thereby reducing catalytic efficiency and signal accuracy.

Addressing this issue will require the development of protective architectures and stimuli-responsive catalytic interfaces. Strategies such as DNA logic gating, protective polymer layers, and selective activation mechanisms may help improve system robustness and operational precision in complex environments.

### Mechanism-guided rational design

Despite rapid progress in experimental demonstrations, a comprehensive theoretical framework describing the structure–activity relationships between DNA regulators and nanozyme catalytic performance is still lacking. Most current systems rely on empirical optimization rather than predictive design, and key mechanisms such as interfacial electron transfer and DNA conformational dynamics remain insufficiently understood.

Future progress will likely rely on integrating advanced characterization techniques with theoretical modeling to reveal catalytic regulation mechanisms at the molecular level. Combining in situ spectroscopy, molecular dynamics simulations, and data-driven material databases may further enable machine learning-assisted rational design of DNA-regulated nanozyme systems.

### Expanding application scenarios

Although current studies mainly focus on biosensing and cancer therapy, the potential applications of DNA-regulated nanozymes remain far from fully explored. Expanding their use in broader biomedical and environmental contexts will be an important direction for future development.

For example, these systems may be applied to neurodegenerative diseases through catalytic clearance of pathological protein aggregates, or to metabolic disorders by compensating for dysfunctional natural enzymes. Moreover, integrating DNA nanozymes with engineered immune cells or adaptive catalytic systems may enable intelligent and feedback-regulated therapeutic platforms. With continued advances in DNA nanotechnology and nanozyme engineering, these programmable catalytic systems are expected to play an increasingly important role in next-generation precision diagnostics and intelligent therapeutics.

## Data Availability

This review draws on data from public peer-reviewed literature, with all supporting original studies cited in the References section.
